# Diffusion of Ions in Phosphonium Orthoborate Ionic Liquids Studied by ^1^H and ^11^B Pulsed Field Gradient NMR

**DOI:** 10.3389/fchem.2020.00119

**Published:** 2020-02-26

**Authors:** Andrei Filippov, Bulat Munavirov, Sergei Glavatskih, Faiz Ullah Shah, Oleg N. Antzutkin

**Affiliations:** ^1^Chemistry of Interfaces, Luleå University of Technology, Luleå, Sweden; ^2^Department of Biological and Medical Physics, Kazan State Medical University, Kazan, Russia; ^3^System and Component Design, KTH Royal Institute of Technology, Stockholm, Sweden; ^4^Department of Electromechanical, Systems and Metal Engineering, Ghent University, Ghent, Belgium; ^5^School of Chemistry, University of New South Wales, Sydney, Australia; ^6^Department of Physics, Warwick University, Coventry, United Kingdom

**Keywords:** nuclear magnetic resonance, ionic liquids, pulsed-field-gradient NMR diffusometry, ^11^B NMR diffusion, ion dynamics

## Abstract

Non-halogenated boron-based ionic liquids (ILs) composed of phosphonium cations and chelated orthoborate anions have high hydrolytic stability, low melting point and exceptional properties for various applications. This study is focused on ILs with the same type of cation, trihexyltetradecylphosphonium ([P_6,6,6,14_]^+^), and two orthoborate anions, such as bis(salicylato)borate ([BScB]^−^) and bis(oxalato)borate ([BOB]^−^). We compare the results of this study with our previous studies on ILs with bis(mandelato)borate ([BMB]^−^) and a variety of different cations (tetraalkylphosphonium, dialkylpyrrolidinium and dialkylimidazolium). The ion dynamics and phase behavior of these ILs is studied using ^1^H and ^11^B pulsed-field-gradient (PFG) NMR. PFG NMR is demonstrated to be a useful tool to elucidate the dynamics of ions in this class of phosphonium orthoborate ILs. In particular, the applicability of ^11^B PFG NMR for studying anions without ^1^H, such as [BOB]^−^, and the limitations of this technique to measure self-diffusion of ions in ILs are demonstrated and discussed in detail for the first time.

## Introduction

Ionic liquids (ILs) are salts that are in a liquid state at temperatures below 100°C. Some ILs are liquids under normal conditions and, thus, are usually called room temperature ionic liquids (RTILs). ILs possess many unique physicochemical properties such as high polarity, non-volatility, high thermal stability, high ionic conductivity, and a wide liquid range, among other properties. Therefore, ILs can be chemically modified and tailored for a wide range of industrial applications (Plechkova and Seddon, 2008; Hallett and Welton, 2011). However, the majority of well-studied ILs consist of halogenated anions such as tetrafluoroborate [BF_4_]^−^ and hexafluorophosphate [PF_6_]^−^, and thus are sensitive to moisture, which limits their utility in many industrial applications. Moreover, halogen-containing ILs may decompose, giving rise to corrosive species such as HF, which can pollute the environment and damage a system in operation (Zhou et al., 2009). Thus, development of non-halogenated and hydrolytically stable ILs for different industrial applications might be an ultimate goal to avoid environmental and health-related issues associated with conventional halogen-containing ILs. For this reason, there is a growing interest in the design of new non-halogenated ILs as an alternative to halogenated ILs during recent years (Shah et al., 2011, 2012, 2013). It has been shown that non-halogenated boron-based ILs composed of tetraalkylphosphonium cations and chelated orthoborate anions have high hydrolytic stability, low melting temperature, outstanding antiwear and friction-reducing properties (Shah et al., 2011, 2013) and high ionic conductivities at elevated temperatures below 100°C (Somers et al., 2013; Shah et al., 2017a,b).

Previously, it was found that the antiwear and friction-reducing performance of various lubricants depends on the molecular motion of the fluidic or semi-fluidic layers between the interacting surfaces. Therefore, local movement and translational diffusion are basic phenomena that must be studied to understand the mechanisms of lubricant performance (Bhushan, 2002; Somers et al., 2013). While there are numerous methods used to study molecular motion, NMR is one of the most informative techniques for measuring local molecular mobility and coefficients of translational diffusion of molecules and ions with magnetically active nuclei (Callaghan, 1991). NMR can be used to study the local (rotational and vibrational) motion of ions and molecules (by examining NMR relaxation times), and the translational motion (using pulse-field-gradient NMR techniques), which are important properties of ions in various applications of ILs.

The usefulness of NMR methods to study ion diffusion in ILs has been validated in a number of previously reported works (Annat et al., 2007; Burrell et al., 2010; Frise et al., 2011; Hayamizu et al., 2011; Filippov et al., 2013, 2014, 2015, 2016, 2018; Taher et al., 2014; Javed et al., 2017; Filippov and Antzutkin, 2018). The diffusion properties of one tetraalkylphosphonium orthoborate-based IL that contains bis(mandelato)borate anions, [P_6,6,6,14_][BMB], has been the most thoroughly investigated (Filippov et al., 2013; Javed et al., 2017). We have previously found that this IL exists in either one or two fluidic “phases” in the temperature range from 20 to 100°C. In a lower temperature range (20–50°C), two phases are present, whereas the cations, [P_6,6,6,14_]^+^, are contained largely in the phase with lower diffusion coefficients, and the anions, [BMB]^−^, are present in the phase with higher diffusion coefficients (Filippov et al., 2013). Laplace NMR methods, combining diffusion and relaxation experiments, revealed additional data about the dynamics and phase structures of [P_6,6,6,14_][BMB] (Javed et al., 2017). Two-dimensional diffusion-relaxation (*D*-*T*_2_) correlation plots have revealed *T*_2_ relaxation times of the slow- and fast-diffusing phases, while 2D *T*_2_-*T*_2_ exchange measurements have quantified the exchange rates of cations and anions between the two phases of [P_6,6,6,14_][BMB] at low temperatures (Javed et al., 2017). Pertinent questions are: (i) whether similar phase-separation properties are present in other orthoborate-based ILs and (ii) whether is there any correlation between the phenomenon of phase separation and the structure of the ions, such as the presence or absence of aromatic or carbonyl chemical groups in orthoborate anions.

The purpose of this work is to further extend studies on two other tetraalkylphosphonium orthoborate-based ILs, i.e., to investigate the bulk mobilities of anions and cations in two orthoborate ILs with the same tetraalkylphosphonium (trihexyltetradecylphosphonium, [P_6,6,6,14_]^+^) cation and two orthoborate anions: bis(salicylato)borate ([BScB]^−^) and bis(oxalato)borate ([BOB]^−^) in the temperature range of 293–363 K. Multinuclear (^1^H and ^11^B) NMR-diffusometry was chosen, because it has been shown previously to be a sensitive method to study any structural as well as dynamic changes in organic molecules and liquid macro- and micro-phases. Moreover, the particular chemical structure of the ILs being studied did not allow sole use of ^1^H NMR, as the [BOB]^−^ anion does not have any hydrogen atoms in its structure. Therefore, complementing a range of our previously reported studies on orthoborate-based ILs, we employed ^1^H and ^11^B NMR to investigate the effect of phenyl- and carbonyl- chemical groups present in orthoborate anions on the dynamics and phase behavior of two selected orthoborate ionic liquids. In addition, to increase our confidence in using a new methodology to study boron-containing ILs, we have also validated the applicability of ^11^B PFG NMR to the previously studied system [P_6,6,6,14_][BMB].

## Experimental Part

### Synthesis and Molecular Organization of the Ionic Liquids

**Figure 1:**
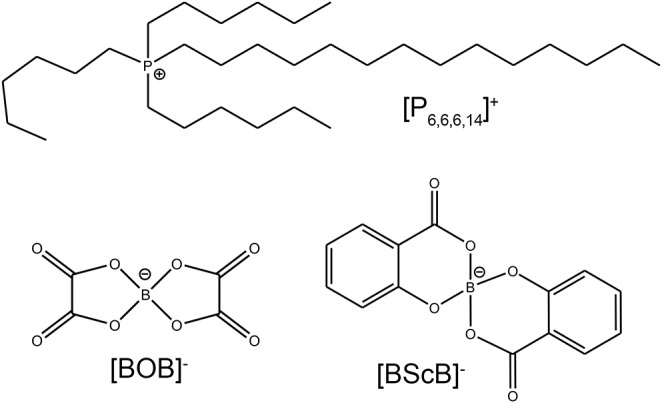
Chemical structures and acronyms of ionic components of the studied phosphonium orthoborate ILs.

The ILs investigated in this study were synthesized and characterized previously by Shah et al. (2011). Chemical structures and abbreviations of the cation and anions in these orthoborate ILs are shown in Figure 1.

The samples are transparent liquids over the entire range of studied temperatures: 293–363 K. Before performing diffusion NMR experiments, each sample was thoroughly degassed in a vacuum oven (*p* < 10^−3^ mbar at T = 333 K) for 50 h. The chemical content of the IL and impurities were checked by liquid ^1^H, ^13^C, ^31^P, and ^11^B NMR and mass-spectrometry and reported earlier (Shah et al., 2011). The thermogravimetric investigation for [P_6,6,6,14_][BMB] revealed high thermal stability, up to 370°C (Shah et al., 2011). Additionally, the repetitive measurements of diffusion coefficients up to 363 K were reproducible, which also confirmed the long-term thermal permanency of this class of orthoborate phosphonium non-halogenated ILs in the temperature range studied.

### Nuclear Magnetic Resonance

NMR spectra of [P6,6,6,14][BScB] were recorded on a Bruker Avance III HD NMR spectrometer with a 11.7 T magnet (Bruker BioSpin AG, Fällanden, Switzerland). Working frequencies were 500.13 MHz for ^1^H and 160.46 MHz for ^11^B. Spectra were recorded using standard Bruker “zg” pulse sequences, spectral width was set to 10 kHz for ^1^H and 200 kHz for ^11^B. Data was processed using Bruker Topspin 4 software. Resonance lines were assigned based on previously published results (Shah et al., 2011, 2012). Probe temperature was calibrated using an external Pt100 thermometer. ^1^H and ^11^B NMR of [P6,6,6,14][BOB] and self-diffusion measurements of the ILs were performed on a Bruker Avance III NMR spectrometer (Bruker BioSpin AG, Fällanden, Switzerland) with Aeon 9.4 T zero-helium boil-off superconducting magnet using a Diff50 (Bruker) Pulsed-Field-Gradient (PFG) probe. Working frequencies were 400.27 MHz for ^1^H and 128.4 MHz for ^11^B. A sample (~300 μl) was placed in a standard glass sample tube (5-mm) and sealed with a plastic plug to avoid interaction with air. Prior to each measurement, the sample was maintained at a certain temperature for 20 min.

The PFG NMR method is one of the most attractive techniques for studying molecular translational motion (Tanner, 1970; Callaghan, 1991). Among the most successful applications of the method is its use in obtaining dynamic and structural information about heterogeneous and multi-component systems such as ionic liquids. With PFG NMR, self-diffusion coefficients of ions can be directly measured. The primary information for the diffusion may be obtained from the diffusion decay (DD) of the NMR spin-echo (SE) or stimulated echo (StE) signal (for details of these methods, see Figures S1, S2). In the SE NMR experiment, after creation of the transverse magnetization by a 90° radiofrequency pulse, nuclear spins are allowed to dephase. At time τ the dephasing process is reversed by the application of a 180° pulse and the nuclear spins begin to rephase and finally they meet together to form a spin-echo at time 2τ. Determination of self-diffusion coefficients of molecules, *Ds*, is accomplished by application of the magnetic field gradient pulses during the dephasing and rephasing period (see Figure S1). These gradients cause the nuclear spins in different positions in the sample to process at different Larmor frequencies. If the spins maintain their positions during the experiment, refocusing proceeds completely into the spin-echo. However, if the spins change their positions, their precession rates will change and the refocusing will be incomplete, which will lead to a decrease in the spin-echo intensity. The time interval 2τ should be comparable with the transverse relaxation time *T*_2_ of the studied nuclei, otherwise, for too short *T*_2_, the intensities of the echo signals will be too small and difficult to detect during a reasonable experimental time frame.

In the stimulated spin-echo experiment (see Figure S2), the diffusion time can be extended to be comparable to the longitudinal relaxation time *T*_1_, which is useful, since in most cases *T*_1_>*T*_2_. Consequently, the dynamic range of the echo decay can be extended as well. However, the intensity of the stimulated echo is half that of the ordinary SE. This property of the StE experiment sets certain limitations, in particular for systems with quadrupolar nuclei and nuclei with low gyromagnetic ratios.

Equations for diffusion of simple, non-associating molecular liquids for SE and StE can be described by Equations (1a) and (1b) (Tanner, 1970; Callaghan, 1991):

(1a) $$\begin{array}{r} A\left(2\tau ,g,\delta \right)=\frac{I}{2}\exp\left(-\frac{2\tau }{T_{2}}\right)\exp\left(-\gamma^{2}\delta^{2}g^{2}Dt_{d}\right) \end{array}$$

(1b) $$\begin{array}{r} A\left(2\tau ,\tau_{1},g,\delta \right)=\frac{I}{2}\exp\left(-\frac{2\tau }{T_{2}}-\frac{\tau_{1}}{T_{1}}\right)\exp\left(-\gamma^{2}\delta^{2}g^{2}Dt_{d}\right) \end{array}$$

Where *A* is the amplitude of the signal, *I* is the factor proportional to the nuclei content in the system; *T*_1_ and *T*_2_ are the spin-lattice and the spin-spin relaxation times, respectively; τ and τ_1_ are time delays in the pulse sequences; γ is the magnetogyric ratio for the nucleus used; *g* and δ are the amplitude and the length of the gradient pulse; *t*_*d*_ = *(Δ-δ*/3) is the time of diffusion; *Δ* = *(τ + τ*_1_*)*; and *D* is the self-diffusion coefficient. The StE pulse sequence was used for ^1^H NMR diffusion measurements (δ = 2 ms, τ = 5 ms, τ_1_ = 20 ms), while the SE pulse sequence was used for ^11^B NMR diffusion measurements (δ = 1 ms, τ = 8 ms). The Pulsed-Field-Gradient amplitude was varied from 0 to 29.8 T/m.

## Results and Discussion

**Figure 2:**
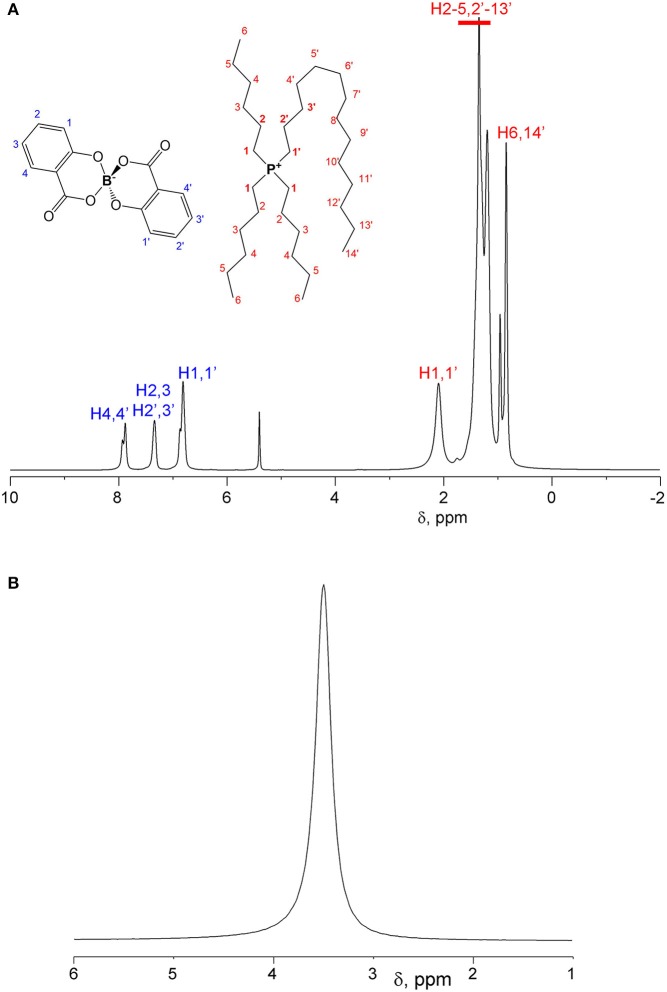
^1^H NMR **(A)** and ^11^B NMR **(B)** spectra of [P_6,6,6,14_][BScB] at T = 296 K.

**Figure 3:**
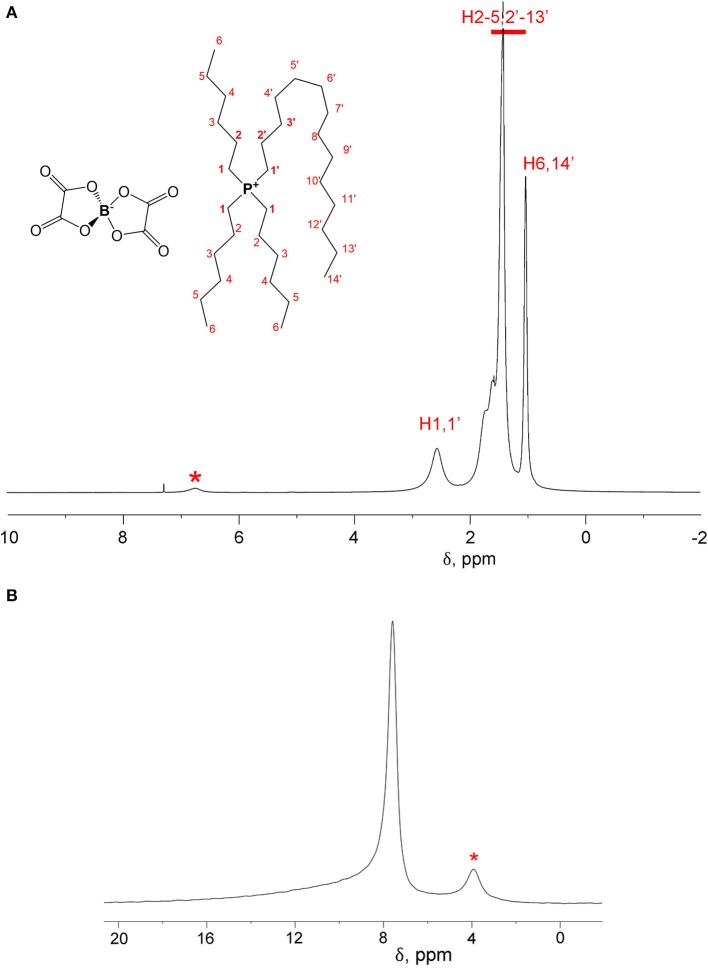
^1^H NMR **(A)** and ^11^B NMR **(B)** spectra of [P_6,6,6,14_][BOB] at T = 296 K. An impurity present in this sample is labelled with a star in both ^1^H and ^11^B NMR spectra.

^1^H and ^11^B spectra of [P_6,6,6,14_][BScB] and [P_6,6,6,14_][BOB] are shown in Figures 2 and 3, respectively. In the case of [P_6,6,6,14_][BScB], ^1^H NMR spectra (Figure 2A) contain resonance lines corresponding to the anion in the aromatic region and NMR signals in the aliphatic range associated exclusively with the cation; all results are in agreement with the previous study (Shah et al., 2011). Only protons of the cation are detected in the ^1^H NMR spectrum of [P_6,6,6,14_][BOB] (Figure 3A), as the [BOB]^−^ anion does not contain protons. At the same time, both [BScB]^−^ and [BOB]^−^ in the named ILs contain boron atoms in the anions and, therefore, can be detected by ^11^B NMR (Figures 2B, 3B).

NMR diffusion experiments were performed on both ^1^H and ^11^B nuclei. In the case of DDs of [P_6,6,6,14_][BScB], a single ^1^H NMR experiment allows diffusion measurements of both cations and anions. At the same time, diffusion of anions can also be measured by ^11^B NMR, because the ^11^B NMR spectrum of this IL contains only a single resonance line from the boron atom in the [BScB]^−^ anion. In the case of [P_6,6,6,14_][BOB], the ^1^H NMR diffusion experiment allows one to measure only diffusion of cations, because of the lack of protons in the [BOB]^−^ anion. Therefore, an additional ^11^B NMR diffusion experiment is necessary to fully characterize diffusion of both cations and anions in [P_6,6,6,14_][BOB].

Diffusion measurements were performed over a wide range of temperatures, from 293 to 363 K. Interestingly, while the ^1^H echo signal was observed in the whole temperature range, the ^11^B NMR echo was observed only in a limited range of temperatures. Moreover, this range was different for each of the ILs in this study. For the [P_6,6,6,14_][BScB] IL, the ^11^B NMR echo was observed only in a low-temperature range, up to 333 K, while for the [P_6,6,6,14_][BOB] IL, the ^11^B NMR echo (SE) signal was observed only in a high-temperature range, between 303 and 363 K. It is suggested that low intensity of ^11^B NMR echoes is caused by the short *T*_2_ relaxation times of quadrupolar boron-11 nuclei (*I* = 3/2) in orthoborate anions in these temperature ranges, specific for each of the ILs studied. Note, that the spin-spin relaxation time *T*_2_ is always smaller (or equal to in some cases) than the spin-lattice relaxation time *T*_1_.

Indeed, one of the main reasons behind the nuclear magnetic relaxation for nuclei having a spin >1/2 is a fluctuating quadrupole interaction between the electric quadrupole moment of nuclei and the local electrostatic field gradient (Hubbard, 1970; Woessner, 2001). In a uniform isotropic environment (bulk fluid), it is commonly assumed that the time dependence of the correlation function for molecular rotational mobility is the exponential decay exp(−|*t*|/τ_*c*_), where τ_*c*_ is the correlation time. Expressions that describe *T*_1_ NMR relaxation of spin 3/2 are the following (Hubbard, 1970; Woessner, 2001):

(2) $$\begin{array}{r} F\left(t\right)=\left(1/5\right)\exp\left(-r_{1}t\right)+\left(4/5\right)\exp\left(-r_{2}t\right) \end{array}$$

(3) $$\begin{array}{r} r_{1}=A\cdot j_{1}\left(\omega_{0}\right) \end{array}$$

(4) $$\begin{array}{r} r_{2}=A\cdot j_{2}\left(2\omega_{0}\right) \end{array}$$

where

(5) $$\begin{array}{r} A=\left(2/5\right)\pi^{2}\left(1+\eta^{2}/3\right){\left(QCC\right)}^{2} \end{array}$$

spectral densities are

(6) $$\begin{array}{r} j_{n}\left(n\omega_{0}\right)=\tau_{c}/\left(1+n^{2}\omega_{0}^{2}\tau_{c}^{2}\right) \end{array}$$

and *QCC* and η are the quadrupole coupling constant and the asymmetry of the quadrupolar tensor, respectively.

**Figure 4:**
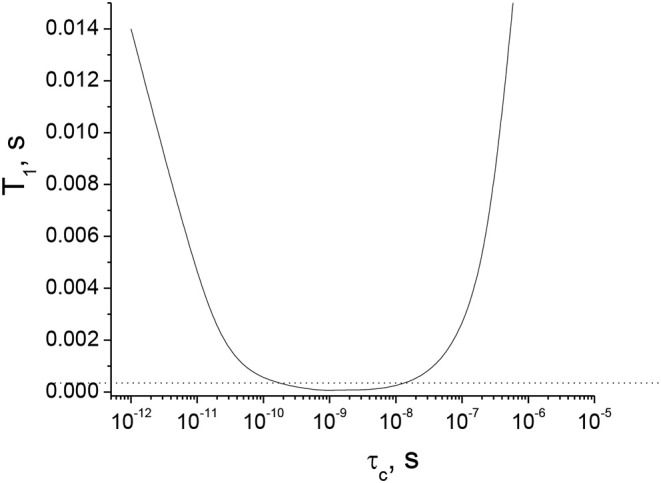
*T*_1_ (^11^B) NMR relaxation time as a function of the correlation time of the rotational mobility, τ_*c*_, based on the quadrupolar interaction in the model of a single isotropic environment (Hubbard, 1970; Woessner, 2001) for an orthoborate anion, calculated using Equations (2)–(6).

*QCC* and η for the ^11^B nucleus in an orthoborate structure trapping an unpaired electron have been calculated in Kordas (2003) to be 2.44 and 0.02 MHz, respectively. A dependence of *T*_1_ on τ_*c*_ was calculated in this work (see Figure 4). As seen from the figure, *T*_1_ decreases below 0.5 ms in the range of τ_*c*_ from 2·10^−10^ to 1·10^−8^ s. Basically, short *T*_1_ limits *T*_2_ and, thus, limits the length of the whole pulse sequence in the diffusion experiment to times shorter than 0.5 ms to observe the NMR signal (diffusion decays). Such short times for diffusion pulse sequences are not technically possible today using standard PFG NMR probes.

Therefore, in the range of τ_*c*_ from 2·10^−10^ to 1·10^−8^ s, the DD ^11^B NMR signal cannot be detected using our PFG NMR probe with the special insert for ^11^B nuclei. We can conclude that the [BScB]^−^ and [BOB]^−^ anions, having different molecular structures, bulkiness and different electrostatic and van der Waals interactions with cations in the studied tetraalkylphosphonium orthoborate ILs, can reach this specific range of correlation times at different temperatures, giving rise to *T*_1_ (and *T*_2_) of <0.5 ms and the ^11^B NMR spin-echo signals of negligibly small intensities.

**Figure 5:**
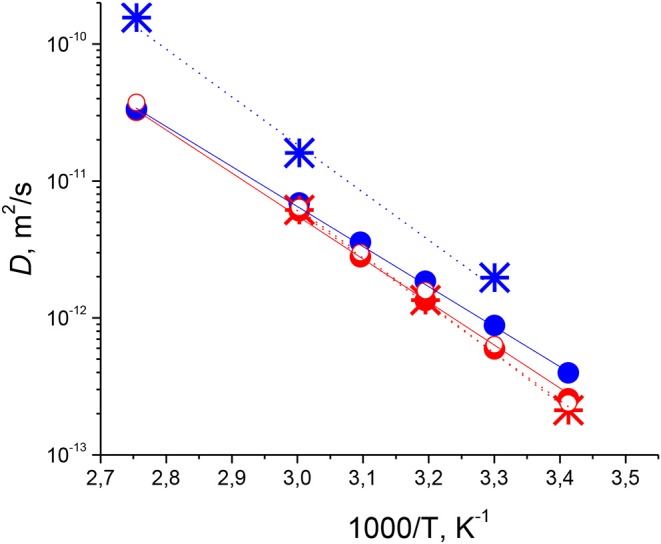
Arrhenius plots of temperature dependences of *Ds* for [P_6,6,6,14_][BScB] (red circles and stars) and [P_6,6,6,14_][BOB] (blue circles and stars). *Ds* of cations determined from ^1^H NMR (solid circles). *Ds* of anions determined from ^1^H NMR (open circles) and from ^11^B NMR (stars).

The obtained temperature dependences of *Ds* of cations and anions for the tetraalkylphosphonium orthoborate ILs are shown in Figure 5.

In Figure 5, we can see that *Ds* of anions determined from ^1^H NMR (red open circles) and ^11^B NMR (stars) determined for [P_6,6,6,14_][BScB] almost coincide with each other, which confirms the credibility of the ^11^B NMR diffusion experiment and validates the concept that ^11^B PFG NMR can be used to determine diffusion coefficients of ions alongside with ^1^H NMR diffusion experiments. We also performed an additional validation of the ^11^B PFG NMR method on a previously studied system, [P_6,6,6,14_][BMB] (see Figure S3), for which diffusion was thoroughly explored using ^1^H and ^31^P PFG NMR (Filippov et al., 2013) and by Laplace NMR methods combining diffusion and relaxation experiments (Javed et al., 2017). Diffusion coefficients previously obtained by ^1^H PFG NMR (StE experiments) for both cations and anions, and *Ds* obtained in this work by ^11^B PFG NMR (SE experiment) for anions coincide in the temperature range from 293 to 323 K, in which the ^11^B NMR spin-echo signal was detectable. Therefore, these experiments on [P_6,6,6,14_][BMB], although they did not bring any new information about diffusion of [BMB]^−^ anions, additionally validate the concept of the usefulness of ^11^B NMR diffusometry for studying orthoborate-based ionic liquids.

Note that ^11^B NMR diffusometry is especially important for [P_6,6,6,14_][BOB], where *Ds* of anions can be determined only from ^11^B NMR due to the lack of protons in this anion. Thus, using a combination of ^1^H and ^11^B NMR diffusion, we were able to fully characterize the diffusivity of ions in the studied tetraalkylphosphonium orthoborate systems.

When it comes to the particular values of self-diffusion coefficients in the studied systems, it is clearly seen from Figure 5 that for [P_6,6,6,14_][BScB], *Ds* of the [BScB]^−^ anion are somewhat larger (by a factor of 1.12–1.18) than *Ds* of the [P_6,6,6,14_]^+^ cation. In the case of [P_6,6,6,14_][BOB], *Ds* of the [BOB]^−^ anion determined from ^11^B NMR are considerably larger (by a factor from 2 to 4 at different temperatures) than *Ds* of the [P_6,6,6,14_]^+^ cation in the whole temperature range studied. *Ds* of cations and anions in [P_6,6,6,14_][BOB] are larger than those in [P_6,6,6,14_][BScB] for the entire temperature range studied. Temperature dependences of *Ds* are almost linear in the Arrhenius plot, with slightly different slopes of these plots for these two ILs. They can be used to estimate activation energies for diffusion using Equation (7) (Atkins and de Paula, 2014):

(7) $$\begin{array}{r} Ds=D^{*}\cdot \exp\left(\frac{-E_{D}}{RT}\right) \end{array}$$

where D^*^ is a parameter that is not dependent on temperature, *E*_*D*_ is the molar activation energy of translational motion, and *R* is the universal gas constant. Results of the analysis are tabulated in Table 1, which demonstrates that the activation energies of diffusion of [P_6,6,6,14_]^+^ cations in [P_6,6,6,14_][BOB] and [P_6,6,6,14_][BScB], and of [BScB]^−^ anions in [P_6,6,6,14_][BScB] are comparable, 57.75 ± 3.25 kJ/mol, while *E*_*D*_ of the [BOB]^−^ anion in the [P_6,6,6,14_][BOB] IL is considerably larger (66.7 kJ/mol), i.e., by 12.2 kJ/mol (*ca* 20%) than the *E*_*D*_ of the [P_6,6,6,14_]^+^ cation in this IL.

**Table 1:** Activation energies for diffusion of phosphonium orthoborate ILs, *E*_*D*_ (in kJ/mol), estimated from the Arrhenius plots of temperature dependences of *Ds* as shown in Figure 5 using Equation (7) and from reference (Filippov et al., 2013) for [P_6,6,6,14_][BMB].

**[P_**6,6,6,14**_][BMB] cations and anions, ^**1**^H and ^**11**^B NMR, “slow” diffusion**	**[P_**6,6,6,14**_][BScB] cations and anions, ^**1**^H and ^**11**^B NMR**	**[P_**6,6,6,14**_][BOB] cations, ^**1**^H NMR**	**[P_**6,6,6,14**_][BOB] anions, ^**11**^B NMR**
58.7	61.0	54.5	66.7

The observed peculiarities of ion diffusion in the phosphonium orthoborate ILs can be discussed from the point of view of a free-volume theory, which was initially proposed by Cohen and Turnbull (1959) and a modified version of this theory proposed by Macedo and Litovitz (1965). This theory considers a particle executing a random walk, while each elementary step of this progression is limited by the occurrence of a free volume larger than a critical size next to the diffusing particle, and by the thermal energy required to perform this step. In this model, diffusion coefficients have the following form:

(8) $$\begin{array}{r} Ds=D^{*}\exp\left(-\frac{\beta \cdot a^{*}}{a_{f}}-\frac{E^{*}}{kT}\right) \end{array}$$

where β is a correction factor for overlapping free volumes (β is in the range from 0.5 to 1), *a*_*f*_ = *a* – a^*^ is the free volume, *a* is an average volume of a molecule in the system, a^*^ is a critical size of the molecular volume (at *a* = a^*^ molecules are packed tightly enough and, therefore, do not diffuse, a^*^ is typically suggested to be the van der Waals volume of a molecule), E^*^ is the energy threshold (activation energy), *k* is the Boltzmann constant and *T* is temperature. Close values of diffusion coefficients of cations and anions and equal energies of activation of these diffusion processes in [P_6,6,6,14_][BScB] may be explained by the fact that these diffusion processes occur in a homogeneous “matrix,” where diffusivities of ions are determined mainly by the “free volume” term of Equation (8). However, a^*^ and *a*_*f*_ for cations and anions in this matrix are different, due to the differences in ions: for smaller size anions a^*^ is slightly smaller, which explains systematically larger values of *Ds* of anions compared to *Ds* of cations in the ionic liquids in this study.

The situation is different when it comes to [P_6,6,6,14_][BOB], where both activation energies and diffusion coefficients are significantly different for anions and cations in this IL, and such differences cannot be explained only from the point of view of different ion sizes. Earlier, a significant difference in diffusion coefficients of anions and cations was observed in the [P_6,6,6,14_][BMB] IL (Filippov et al., 2013), with a clear phase separation phenomenon at temperatures lower than ca. 313 K (see also Figure S3). A pertinent question is whether the significant difference in values of cations and anions observed for [P_6,6,6,14_][BOB] (see Figure 5, Table 1) is also related to a phase separation in this IL. A closer look, however, suggests that in the case of [P_6,6,6,14_][BOB], two different *Ds* and *E*_*D*_ correspond to two different ions (i.e., to cations [P_6,6,6,14_]^+^ and anions [BOB]^−^). Thus, a plausible explanation would be that each ion diffuses in its own micro- or nano-phase and the two bi-continuous (sponge-like) phases should be intercalated to fulfill electroneutrality of the whole system, as was previously observed in ammonium tetrafluoroborate IL (Frise et al., 2011). Values of *Ds* for cations and anions in [P_6,6,6,14_][BOB] IL are significantly larger than those in the [P_6,6,6,14_][BScB] IL, which could be explained by looser packing of ions in both bi-continuous micro-phases of [P_6,6,6,14_][BOB] in comparison to the packing of ions in a homogeneous one-liquid phase [P_6,6,6,14_][BScB] IL in the whole temperature range studied. The discussed differences in ionic mobilities are obviously correlated with differences in van der Waals interactions between anions and cations in these two selected ILs: strong attractive forces between hydrophobic phenyl groups in [BScB]^−^ anions and alkyl-groups in [P_6,6,6,14_]^+^ cations in the [P_6,6,6,14_][BScB] IL vs. significantly weaker attractive forces between four (and the only) hydrophilic carbonyl groups in [BOB]^−^ anions and alkyl-groups in [P_6,6,6,14_]^+^ cations in the [P_6,6,6,14_][BOB] IL. However, molecular dynamics simulations should be performed to understand further the suggested correlations between structure of ions, their interactions and mobilities in orthoborate-based ILs.

## Conclusions

In this work, diffusion of ions is measured in two selected tetraalkylphosphonium orthoborate based ILs with the trihexyl(tetradecyl)phosphonium cation in combination with two different orthoborate anions having different chemical groups (phenyl vs. carbonyl) in the temperature range 293–363 K using ^1^H and ^11^B NMR data. In the case of the [P_6,6,6,14_][BScB] IL, it is shown that the measurements on both types of nuclei give the same values for diffusivity of the [BScB]^−^ anion, which is a convincing validation of usefulness of the ^11^B PFG NMR method to study systems lacking protons (and other) NMR-active nuclei. Additional validation of the ^11^B PFG NMR on a previously studied [P_6,6,6,14_][BMB] IL using ^1^H PFG NMR further confirmed the reliability of the method. Furthermore, application of ^1^H and ^11^B NMR allowed us to fully characterize diffusion of ions in these systems. Combined ^1^H and ^11^B NMR diffusion data demonstrates that [P_6,6,6,14_][BScB] forms a homogeneous liquid phase in the whole studied temperature range. In contrast, in the case of [P_6,6,6,14_][BOB], for which only ^11^B NMR diffusion can be used for measuring the diffusion coefficient of the [BOB]^−^ anion, which lacks protons, micro- or nano-phase separation of ions into two different bicontinuous sub-phases is revealed, in which self-diffusion of [P_6,6,6,14_]^+^ cations and [BOB]^−^ anions, moving in their own micro-phases, is characterized by different diffusion coefficients and different activation energies of diffusion. Thus, a multinuclear NMR diffusion technique is demonstrated here as a useful tool to identify different types of phase phenomena in ionic liquids.

## Data Availability Statement

The datasets generated for this study are available on request to the corresponding author.

## Author Contributions

All authors listed have made a substantial, direct and intellectual contribution to the work, and approved it for publication.

### Conflict of Interest

The authors declare that the research was conducted in the absence of any commercial or financial relationships that could be construed as a potential conflict of interest.

## References

[B1] Annat G. MacFarlane D. R. Forsyth M. (2007). Transport properties in ionic liquids and ionic liquis mixtures: the challenges of NMR pulsed field gradient diffusion measurements. J. Phys. Chem. B 111, 9018–9024. 10.1021/jp072737h 17608524

[B2] Atkins P. de Paula J. (2014). Atkin's Physical Chemistry. Oxford: Oxford University Press.

[B3] Bhushan B. (2002). Introduction to Tribology. New York, NY: Willey & Sons.

[B4] Burrell G. L. Burgar I. M. Gong Q. Dunlop N. F. Separovič F. (2010). NMR relaxation and self-diffusion study at high and low magnetic fields of ionic association in protic ionic liquids. J. Phys. Chem. B 114, 11436–11443. 10.1021/jp105087n 20712307

[B5] Callaghan P. T. (1991). Principles of Nuclear Magnetic Resonance Microscopy. Clarendon, TX: Oxford.

[B6] Cohen M. Turnbull D. (1959). Molecular transport in liquids and glasses. J. Chem. Phys. 31, 1164–1169. 10.1063/1.1730566

[B7] Filippov A. Antzutkin O. N. (2018). Magnetic field effects dynamics of ethylammonium nitrate ionic liquid confined between glass plates. Phys. Chem. Chem. Phys. 20, 6316–6320. 10.1039/C7CP06554J 29435522

[B8] Filippov A. Azancheev N. Gibaydullin A. Bhattacharyya Sh. Antzutkin O. N. Shah F. U. (2018). Dynamic properties of imidazolium orthoborate ionic liquids mixed with polyethylene glycol studied by NMR diffusometry and impedance spectroscopy. Magn. Reson. Chem. 56, 113–119. 10.1002/mrc.4636 28752526

[B9] Filippov A. Azancheev N. Shah F. U. Glavatskih S. Antzutkin O. N. (2016). Self-diffusion of phosphonium bis(salicylato)borate ionic liquid in pores of Vycor porous glass. Micropor. Mesopor. Mater. 230, 128–134. 10.1016/j.micromeso.2016.04.044

[B10] Filippov A. Azancheev N. Taher M. Shah F. U. Rabét P. Glavatskih S.. (2015). Self-diffusion and interactions in mixtures of imidazolium bis(mandelato)borate ionic liquids with poly(ethylene glycol): ^1^H NMR study. Magn. Reson. Chem. 53, 493–497. 10.1002/mrc.4232 25854162

[B11] Filippov A. Shah F. U. Taher M. Glavatskih S. Antzutkin O. N. (2013). NMR self-diffusion study of a phosphonium bis(mandelato)borate ionic liquid. Phys. Chem. Chem. Phys. 15, 9281–9287. 10.1039/c3cp51132d 23661052

[B12] Filippov A. Taher M. Shah F.-U. Glavatskih S. Antzutkin O.N. (2014). The effect of the cation alkyl chain length on density and diffusion in dialkylpyrrolidinium bis(mandelato)borate ionic liquids. Phys. Chem. Chem. Phys. 16, 26798–26805. 10.1039/C4CP03996C 25372279

[B13] Frise A. E. Ichikawa T. Yoshio M. Ohno H. Dvinskikh S. V. Kato T.. (2011). Ion conductive behaviour in a confined nanostructure: NMR observation of self-diffusion in a liquid-crystalline bicontinuous cubic phase. Chem. Commun. 46, 728–730. 10.1039/B915931B 20087501

[B14] Hallett J. Welton T. (2011). Room-temperature ionic liquids: solvents for synthesis and catalysis, 2. Chem. Rev. 111, 3508–3576. 10.1021/cr1003248 21469639

[B15] Hayamizu K. Tsuzuki S. Seki S. Umebayashi Y. (2011). Nuclear magnetic resonance studies on the rotational and translational motions of ionic liquids composed of 1-ethyl-3-methylimidazolium cation and bis(trifluoromethanesulfonyl)amide and bis(fluorosulfonyl)amide anions and their binary systems including lithium salts. J. Chem. Phys. 135:084505. 10.1063/1.3625923 21895197

[B16] Hubbard P. S. (1970). Nonexponential nuclear magnetic relaxation by quadrupole interactions. J. Chem. Phys. 53, 985–987. 10.1063/1.1674167

[B17] Javed M. A. Ahola S. Håkansson P. Mankinen O. Aslam M. K. Filippov A.. (2017). Structure and dynamics elucidation of ionic liquids by multidimensional Laplace NMR. Chem. Commun. 53, 11056–11059. 10.1039/C7CC05493A 28948273

[B18] Kordas G. (2003). Equivalent exploitation of four-pulse one-dimensional ESEEM and HYSCORE spectroscopies for the elucidation of BOHC defects in borate glasses supported by quantum mechanical calculations. Phys. Rev. 68:024202 10.1103/PhysRevB.68.024202

[B19] Macedo P. B. Litovitz T. A. (1965). On the relative roles of free volume and activation energy in the viscosity of liquids. J. Chem. Phys. 42, 245–256. 10.1063/1.1695683

[B20] Plechkova N. Seddon K. (2008). Applications of ionic liquids in the chemical industry. Chem. Soc. Rev. 37, 123–150. 10.1039/B006677J 18197338

[B21] Shah F. U. Glavatskih S. Antzutkin O. N. (2013). Boron in tribology: from borates to ionic liquids. Tribol. Lett. 51, 281–301. 10.1007/s11249-013-0181-3

[B22] Shah F. U. Glavatskih S. Dean P. M. MacFarlane D. R. Forsyth M. Antzutkin O. N. (2012). Halogen-free chelated orthoborate ionic liquids and organic ionic plastic crystals. J. Mater. Chem. 22, 6928–6938. 10.1039/c2jm12657e

[B23] Shah F. U. Glavatskih S. MacFarlane D. R. Somers A. Forsyth M. Antzutkin O. N. (2011). Novel halogen-free chelated orthoborate-phosphonium ionic liquids: synthesis and tribophysical properties. Phys. Chem. Chem. Phys. 13, 12865–12873. 10.1039/c1cp21139k 21687897

[B24] Shah F. U. Gnezdilov O. I. Filippov A. (2017b). Ion dynamics in halogen-free phosphonium bis(salicylato)borate ionic liquid electrolytes for lithium-ion batteries. Phys. Chem. Chem. Phys. 19, 16721–16730. 10.1039/C7CP02722B 28621370

[B25] Shah F. U. Gnezdilov O. I. Gusain R. Filippov A. (2017a). Transport and association of ions in lithium battery electrolytes based on glycol ether mixed with halogen-free orthoborate ionic liquid. Sci. Rep. 7:16340. 10.1038/s41598-017-16597-7 29180739 PMC5703989

[B26] Somers A. E. Howlett P. C. MacFarlane D. R. Forsyth M. (2013). A review of ionic liquid lubricants. Lubricants 1, 3–21. 10.3390/lubricants1010003

[B27] Taher M. Shah F. U. Filippov A. de Baets P. Glavatskih S. Antzutkin O. N. (2014). Halogen-free pyrrolidinium bis(mandelato)borate ionic liquids: some physicochemical properties and lubrication performance as additives to polyethylene glycol. RSC Adv. 4, 30617–30623. 10.1039/C4RA02551B

[B28] Tanner J. E. (1970). Use of the stimulated echo in NMR diffusion studies. J. Chem. Phys. 52, 2523–2526. 10.1063/1.1673336

[B29] Woessner D. E. (2001). NMR relaxation of spin-3/2 nuclei: effects of structure, order, and dynamics in aqueous heterogeneous systems. Concepts Magn. Reson. 13, 294–325. 10.1002/cmr.1015

[B30] Zhou F. Liang Y. Liu W. (2009). Ionic liquid lubricants: designed chemistry for engineering applications. Chem. Soc. Rev. 38, 2590–2599. 10.1039/b817899m 19690739

