# Supplementary material for: Diffusion of Ions in Phosphonium Orthoborate Ionic Liquids Studied by 1H and 11B Pulsed Field Gradient NMR
Source: Front Chem. 2020 Feb 26;8:119. doi: 10.3389/fchem.2020.00119 (PMC7057796; doi:10.3389/fchem.2020.00119)
Supplement: Supplementary file 1 [file Data_Sheet_1.docx]

**Electronic Supplementary Information**

**Diffusion of ions in phosphonium orthoborate ionic liquids studied by ^1^H and ^11^B pulsed field gradient NMR**

**Andrei Filippov,^a,b^* Bulat Munavirov,^c^ Sergei Glavatskih,^c,d,e^ Faiz Ullah Shah,^a^ and Oleg N. Antzutkin^a,f^**

^a^Chemistry of Interfaces, Luleå University of Technology, 971 87 Luleå, Sweden

^b^Kazan State Medical University, 420012 Kazan, Russia

^c^System and Component Design, KTH Royal Institute of Technology, SE-10044 Stockholm, Sweden

^d^Department of Electromechanical, Systems and Metal Engineering, Ghent University, B-9000 Ghent, Belgium

^e^School of Chemistry, University of New South Wales, Sydney, Australia

^f^Department of Physics, Warwick University, CV4 7AL, Coventry, U.K.

***Corresponding authors**: [andrei.filippov@ltu.se](mailto:andrei.filippov@ltu.se)

Tel: +46 (0)73-6782225


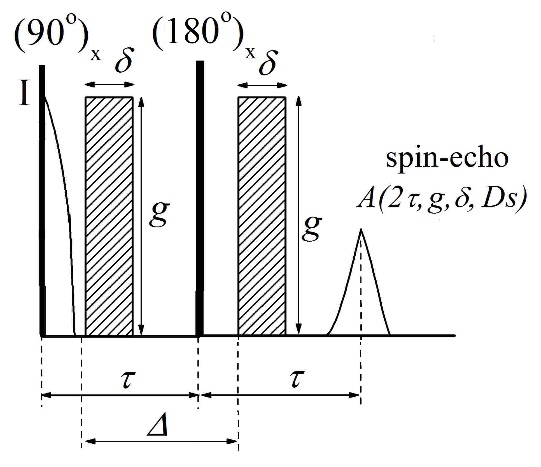


**Figure S1.** Spin-echo pulse sequence for self-diffusion measurements.^1,2^ In this figure, 90^o^ and 180^o^ radiofrequency (RF) pulses are shown as solid bars. Gradient pulses with amplitude *g* and duration *δ* are shown as hatched rectangles. *τ* is the time interval between the first and the second RF pulses, *Δ* is the time between gradient pulses. The observation time of the diffusion process (diffusion time) is *t_d_* = (*Δ* - *δ*/3).


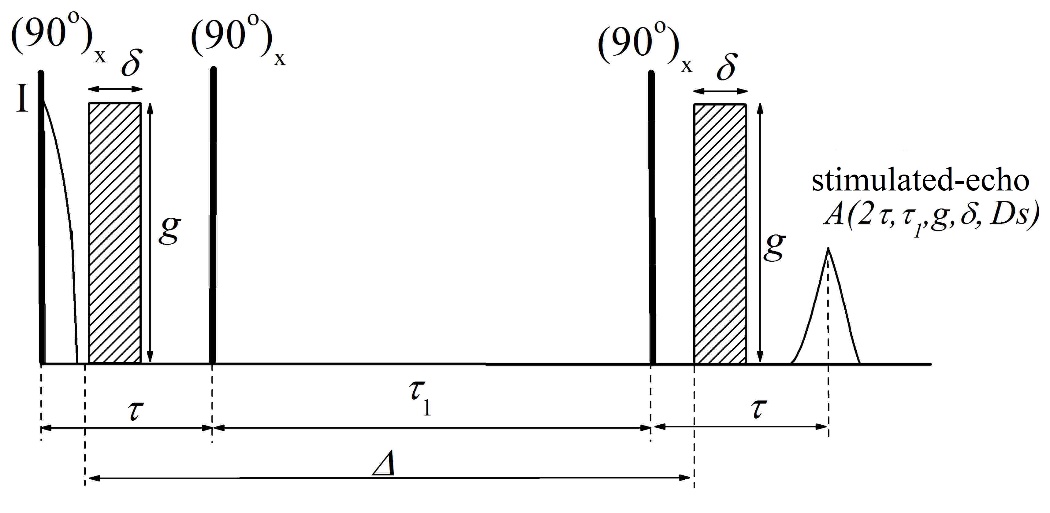


**Figure S2.** Stimulated spin-echo pulse sequence for self-diffusion measurements.^1,2^ In this figure, 90^o^ radiofrequency (RF) pulses are shown as solid bars. Gradient pulses with amplitude *g* and duration *δ* are shown as hatched rectangles. *τ* is the time interval between the first and the second RF pulses; *τ_1_* is the time interval between the second and the third RF pulses; *Δ* is the time between gradient pulses. The observation time of the diffusion process (diffusion time) is *t_d_* = (*Δ* - *δ*/3).

**Figure S3.** Arrhenius plot of *Ds* for “slow-diffusing” and “fast-diffusing” components of [P_6,6,6,14_][BMB]. *Ds* of cations (solid circles) and anions (open circles) have been determined from diffusion decays in ^1^H PFG NMR experiments (data adapted from ref. [3]). *Ds* of anions (red stars) were determined from DDs in ^11^B PFG NMR experiments in this work. Note that only *Ds* of anions in the “slow-diffusing” phase of [P_6,6,6,14_][BMB] were determined in the latter experiment, while *Ds* of anions in the “fast-diffusing” phase of this IL were not observed by ^11^B spin-echo NMR. The latter fact can be explained by the fast relaxation of corresponding ^11^B nuclei and a small relative fraction of the “fast-diffusing” phase and, therefore, vanishingly small amplitudes of DDs in ^11^B PFG NMR experiments (see Figure S4).

**Figure S4.** Temperature dependence of the spin-spin relaxation time, *T_2_*, of ^11^B nuclei in anions of [P_6,6,6,14_][BMB]. *T_2s_* (solid circles) and *T_2f_* (open circles) denote *T_2_* (^11^B) of boron-11 spins in the “slow-diffusing” and the “fast-diffusing” components, respectively. Note that in this study *T_2s_* is changing in the range of 4-6 ms in the temperature interval from 293 to 313 K, while *T_2f_* varies between 1.2 and 1.5 ms in the whole temperature range of 293-333 K. The short *T_2f_* of ^11^B nuclei in anions moving in the “fast-diffusing” phase of [P_6,6,6,14_][BMB] results in small amplitudes (and a poor signal-to-noise ratio) of DDs, measured with the shortest possible diffusion times (*Δ* = 4.7ms) in ^11^B PFG NMR that, in turn, sets limitations on measurements of *Ds* (therefore, this data is absent in Fig. S3).

**References**

1 P. T. Callaghan, *Principles of Nuclear Magnetic Resonance Microscopy*, Clarendon, Oxford, 1991.

2 J. E. Tanner, *J. Chem. Phys*., 1970, **52**, 2523.

3 A. Filippov, F. U. Shah, M. Taher, S. Glavatskih and O. N. Antzutkin, *Phys. Chem. Chem. Phys.*2013, **15**,9281-9287.
